# Notch signaling in diabetic kidney disease: recent progress

**DOI:** 10.3389/fendo.2025.1537769

**Published:** 2025-07-31

**Authors:** Zhi-Hui Wang, Wei Tu, Ya-Ni Long, Peng-Fei Li, Kai-Ying He, Jing Wu

**Affiliations:** ^1^ First Clinical Medical College, Fujian University of Traditional Chinese Medicine, Fujian, Fuzhou, China; ^2^ Department of Dermatology, The Second Affiliated Hospital of Chongqing Medical University, Chongqing, China; ^3^ Department of Tumor and Endocrinology, The Affiliated Chinese Medicine Hospital of Chongqing Three Gorges Medical College, Chongqing, China; ^4^ Department of Nephrology, People’s Hospital Affiliated to Fujian University of Traditional Chinese Medicine, Fuzhou, China

**Keywords:** Notch signaling, diabetic kidney disease, renal fibrosis, podocyte, therapy

## Abstract

Diabetic kidney disease (DKD) is one of the most serious complications of diabetes mellitus (DM) and the main cause of end-stage renal disease (ESRD). The number of affected patients is increasing annually worldwide. Therefore, it is necessary to establish new strategies to treat DKD and improve prognosis. The Notch signaling pathway is involved in multiple mechanisms in DKD, including glomerular endothelial dysfunction, filtration barrier damage, podocyte EMT and dedifferentiation, tubulointerstitial fibrosis, proximal tubule cell dedifferentiation, macrophage polarization, etc. In addition, Notch signaling interacts with other pathways involved in DKD progression, such as TGF-β, Wnt/β-catenin, mTOR, AMPK, autophagy, etc. Therefore, new ideas for the future treatment of DKD may be provided through clarification of the role of the Notch signaling pathway and development of novel drugs.

## 1 Introduction

In recent years, the incidence of diabetes mellitus has increased dramatically. According to estimates, the prevalence of diabetes among people aged 20 to 79 worldwide was 10.5% in 2021 and will increase to 12.2% in 2045, which represents an increase of over 50% compared to 2017 levels (1, 2). Diabetic kidney disease is one of the most serious complications of diabetes and the main cause of end-stage renal disease worldwide (3). The typical clinical course of DKD begins with microalbuminuria, followed by severe proteinuria, which in turn induces tubular damage, progressing from low-grade renal inflammation to renal fibrosis, renal sclerosis, and finally to end-stage renal disease (4). When DKD develops to the ESRD stage, dialysis or kidney transplantation is essential. Diabetic kidney disease is prevalent worldwide, causing serious health problems and huge economic burdens on global human society (5). The pathogenesis of DKD involves multiple aspects and multiple signals. Notch signaling is involved in the pathogenesis of DKD, including vascular endothelial disorders (6), renal inflammation (7), renal fibrosis and necrosis (8), and podocyte and tubular epithelial cell damage (9). This article reviews the role of the Notch signaling pathway in DKD and discusses the molecular mechanism of Notch signaling pathway regulation. It is envisioned that analysis of the functional significance of Notch signaling will be critical to the development of novel therapeutic approaches for DKD.

## 2 Notch signaling pathway

Notch signaling has a role in many facets of metazoan life, such as cell fate determination, embryonic development, tissue repair and function, and non-cancerous and malignant disorders (10). Multiple intermediates exist between the nuclear effectors and membrane receptors in classical Notch signaling pathways, which are mediated by G protein-coupled receptors (GPCRs) and enzyme-linked receptors (11, 12). The Notch signal is transmitted between adjacent cells via the Notch receptor, which undergoes three cleavages and translocates to the nucleus to regulate the transcription of target genes (13). Mammals, including humans, are known to possess four distinct Notch receptors: Notch 1, 2, 3, and 4 (14). Delta-like ligand 1 (DLL1), delta-like ligand 3 (DLL3), delta-like ligand 4 (DLL4), Jagged-1 (JAG1), and Jagged-2 (JAG2) are the five recognized Notch ligands in humans (15, 16). Each of these ligands performs both redundant and distinct roles. DLL1 is in charge of cell differentiation and cell-to-cell communication (16), DLL3 induces apoptosis to stop cell growth (17), DLL4 activates NF-κB signaling to improve tumor metastasis and VEGF secretion (18), JAG1 stimulates angiogenesis, and JAG2 encourages cell survival and proliferation (16). Notch signaling are transmitted via the binding of Notch receptors and ligands, which are likewise single-transmembrane proteins produced on the cell surface.

**Figure 1:**
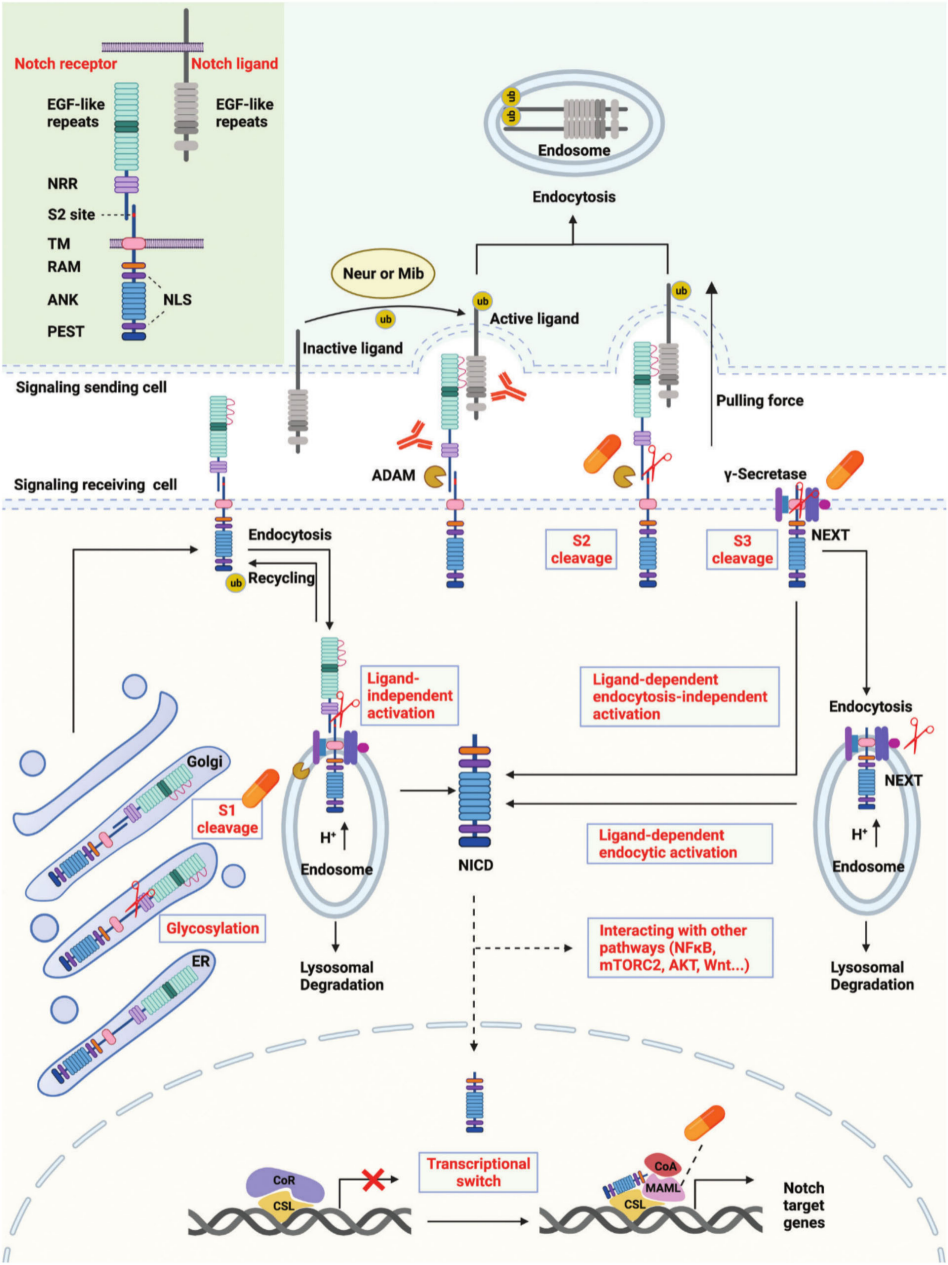
Overview of the Notch signaling pathway and pivotal targets.

In cells receiving signals, Notch receptors are first produced in the endoplasmic reticulum (ER) and then transported to the Golgi apparatus. The EGF-like repeat domain of Notch receptors is glycosylated during transport. The Notch receptors are then split into heterodimers (S1 cleavage) in the Golgi apparatus and moved to the cell membrane. Certain Notch receptors on the cell membrane are incorporated into endosomes with the aid of ubiquitin ligases (19). Metalloproteases (ADAMs) and γ-secretase are found in an acidic environment inside endosomes. Endosome notch receptors can be broken down in lysosomes, returned to the cell membrane, or cleaved into NICD. The portion of the Notch receptor that remains after S2 cleavage is known as Notch extracellular truncation (NeXT) (20–22). NeXT can be endocytosed into endosomes or further broken down by γ-secretase on the cell membrane. NICD is released onto the cell membrane in the former way. In the latter mode, NeXT can be taken to lysosomes, where it will be degraded, or it can be broken down into NICD. In general, there are three pathways for the production of NICD, namely ligand-independent activation, ligand-dependent endocytosis-independent activation, and ligand-dependent endocytosis-activated (10). Interactions between NICD and several signaling pathways, including NF-κB, mTORC2, AKT, and WNT, are mediated by its translocation into the nucleus or retention in the cytoplasm. According to the traditional theory, CBF-1/suppressor of hairless/Lag1 CSL binds to the corepressor and prevents target gene transcription in the absence of NICD (23–25). After binding to CSL and enlisting Mastermind-like proteins (MAML), NICD can reach the nucleus and promote the transcription of Notch target genes by releasing co-repressors and enlisting co-activators (**Figure 1**).

## 3 Glomerulus dysfunction in typical DKD and Notch

Diabetic kidney disease is a primary microvascular complication of diabetes (26, 27). However, the exact pathways mediating endothelial dysfunction in DKD are poorly understood. Glomerular endothelial cells (GEnCs) are specialized vascular cells found in the kidney that serve as the walls of the glomerular tufts and are crucial for maintaining renal homeostasis. The glycocalyx, a network of endothelial polysaccharide layers, covers GEnCs. In animal studies, the loss of the glycocalyx is correlated with the severity of proteinuria (28). Endothelial cell dysfunction increases endothelial permeability and apoptosis and can lead to proteinuria. The Notch signaling pathway is involved in the regulation of the glomerular filtration barrier. Endothelial Notch1 signaling activation downregulates VE-cadherin levels through transcription factors SNAI1 and ERG, reducing glomerular endothelial glycocalyx, thereby leading to glomerular filtration barrier dysfunction and proteinuria (29). The Notch pathway plays a crucial role in kidney development, including guiding the differentiation of various progenitor cells, and abnormal Notch signaling leads to severe changes in cell fate (30). Endothelial ADAM10, a key regulator of Notch signaling, promotes the development and maturation of glomerular vasculature (31).

One of the main characteristics of diabetes complications is abnormal angiogenesis. Podocyte-released vascular endothelial growth factor A (VEGFA) attaches to its receptors, VEGFR1 and VEGFR2, which are expressed on GEnCs. VEGFA stimulates sprouting angiogenesis and regulates GEnC activity. Notch signaling interacts with other key factors, such as VEGF-A, to affect the health status and pathological processes of glomeruli (32, 33). The increase of Notch1 signaling in renal podocytes treated with high glucose causes VEGF production, which in turn causes nephrin inhibition and apoptosis (34). In diabetic kidneys, Notch1 knockdown results in decreased proteinuria, decreased nephrin expression, and decreased VEGF expression. The pathophysiology of endothelial dysfunction in diabetic nephropathy and retinopathy may involve Notch signaling. By modulating the susceptibility of hemangioblasts to VEGF, Notch signaling does prevent diabetic extrarenal angiogenesis (35, 36). Activation of Notch signaling in endothelial cells can promote neovascularization and increase microvascular permeability, destroy adhesion junctions between endothelial cells, mediate endothelial cell dysfunction, and eventually lead to diabetic endothelial cell dissociation (37). Activation of the Notch1 signaling pathway can cause endothelial cell leakage, damage the glomerular filtration barrier, and lead to increased urinary protein (38). Low-intensity pulsed ultrasound-induced calcium influx promotes the beneficial effects of angiogenesis, improved renal function, and Akt-eNOS phosphorylation in rats with acute kidney injury through the Notch1-Akt-eNOS signaling pathway, making Notch1 activation a therapeutic strategy for acute kidney injury targeting angiogenesis (39). Endothelial-mesenchymal transition (EndMT) is a hallmark of diabetes-related vascular complications. Intermittent high glucose exposure upregulates H3K4me3 levels in glomerular endothelial cells of Ob/Ob mice, activates Notch signaling, and induces some mesenchymal-like features in endothelial cells (40). Through Notch activation, matrix metalloproteinase-9 causes the endothelium-mesenchymal transition in human glomerular endothelial cells (41).

Podocytes are terminally differentiated cells that are unable to proliferate. Foot process effacement and podocyte hypertrophy are observed in early and middle stages of DKD, while late stages of the disease are characterized by podocyte death and dedifferentiation. An irreversible stage in the pathophysiology of DKD is the loss of more than 20% of podocytes, which results in glomerular scarring and the onset of end-stage renal disease (42, 43). In DKD, podocyte injury is a key event leading to proteinuria, nephropathy, glomerulosclerosis, and loss of renal function (44). The Notch signaling pathway plays an important role in glomerular cells, especially podocytes (32). Studies have shown that activation of Notch signaling can promote glomerular lesions and albuminuria by regulating TGF-β expression and activity (45). High-level activation of the Notch1 signaling pathway weakens the improvement effect of islet transplantation on kidney damage and the recovery of podocytes. The use of N-[N-(3,5-difluorophenacetyl)-L-alanyl]-S-phenylglycine t-butyl ester (DAPT) can inhibit the excessive activation of the Notch1 pathway and improve podocyte damage under high glucose conditions (46). A study done in a lab setting found that microencapsulated islet transplantation reduced the levels of Jag-1, Notch1, and Hes-1 proteins in rat glomeruli. It also stopped Notch signaling and improved damage to podocytes in a high glucose environment (47). Abnormal activation of the Notch1 signaling pathway may affect the therapeutic effect of islet transplantation in diabetic nephropathy by changing the balance of podocyte apoptosis and autophagy (46). Under high glucose conditions, the reduction of Sirt6 leads to overactivation of Notch signaling, resulting in damage to podocytes, including inflammation, apoptosis, and reduced autophagy levels. Sirt6 deacetylates H3K9, inhibiting the transcription of Notch1 and Notch4 genes, thereby protecting podocytes from damage (48). Under hyperglycemia, MAD2B expression is upregulated in diabetic glomeruli and cultured podocytes. Upregulated MAD2B expression can lead to Numb loss and activation of the Notch1 signaling pathway during the progression of DKD, ultimately leading to podocyte injury. Podocyte-specific deletion of MAD2B can alleviate podocyte injury and renal function deterioration in diabetic nephropathy mice (49). High glucose-induced CDKN2B-AS1 promotes apoptosis and fibrosis in human podocytes and human tubular cells through the TGF-β1 signaling pathway mediated by the miR-98-5p/NOTCH2 axis (50).

**Figure 2:**
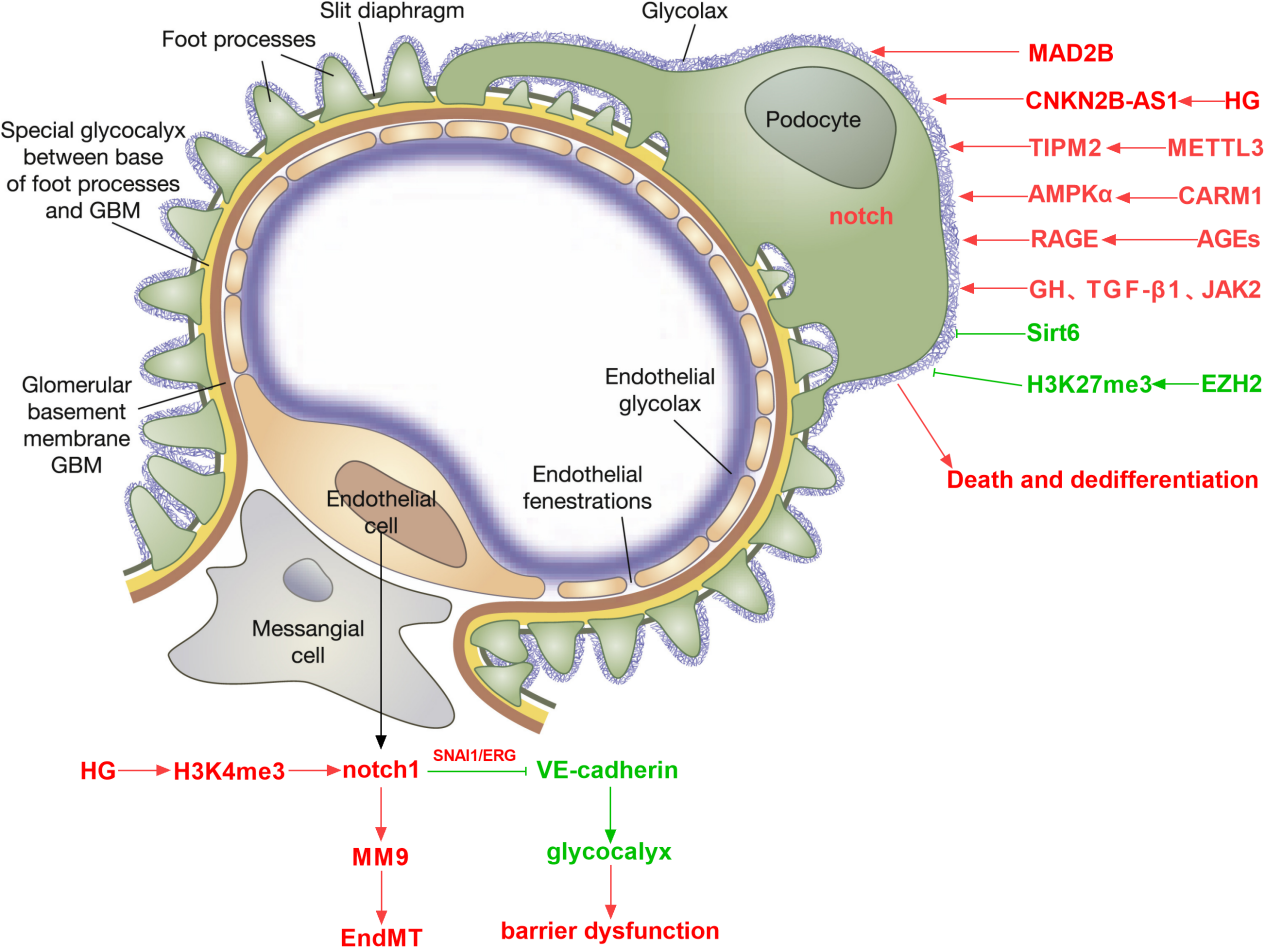
Role of Notch signaling in regulating DKD-associated endothelial cell dysfunction, podocyte dedifferentiation, and death.

Research revealed that removing the histone methylating enzyme EZH2 from podocytes reduced the levels of H3K27me3 and made animals more susceptible to glomerular illness. H3K27me3 was enriched at the promoter region of the Notch ligand Jag1 in podocytes, and derepression of Jag1 by EZH2 inhibition facilitated the activation of Notch signaling and podocyte dedifferentiation (51). METTL3 affects the stability of TIMP2 mRNA by regulating the m6A modification level, thereby enhancing the expression of TIMP2. The increase in TIMP2 further activates the Notch signaling pathway, promoting the expression of inflammatory factors and podocyte apoptosis. TIMP2 participates in the progression of diabetic nephropathy by regulating the Notch signaling pathway (52). A CARM1-AMPKα-Notch1-CB1R signaling axis mediates the high-glucose-induced podocyte apoptosis. In DKD, podocyte loss may be avoided by employing techniques to maintain CARM1 expression or lower the enzymatic activity of a ubiquitin ligase specific for CARM1 (53). In DKD, advanced glycation end products (AGEs) can damage podocytes through the Notch1 signaling pathway. AGEs directly damage podocytes through the RAGE-Notch1 signaling pathway and cytoskeletal remodeling, promoting epithelial-mesenchymal transition (EMT) and functional loss of podocytes. Notch1 signals activated by AGEs also promote podocyte mesenchymalization, leading to collagen deposition, disappearance of foot processes, and renal tubular lesions, which in turn induce proteinuria (54). Notch signaling plays a pivotal role in podocyte EMT and is intricately connected with other pathways like Wnt and TGF-β. Under hyperglycemia, Notch activation crosses with TGF-β, Wnt, and other pathways to promote the EMT process of podocytes, causing podocytes to lose their unique epithelial properties, leading to proteinuria and the progression of glomerular diseases (55). Notch signaling promotes the initiation of the cell cycle by transmitting short-range signals between cells. GH and TGF-β1 induce podocytes to re-enter the cell cycle from the quiescent phase (G0 phase) by activating the Notch signaling pathway, but these cells are abnormal when completing mitosis, showing that only karyokinesis but not cytokinesis is completed, causing the cells to become binuclear and eventually undergo mitotic catastrophe, ultimately causing podocyte death (56). Inhibition of JAK2, TGFBR1, or Notch signaling pathways can prevent re-entry of the cell cycle and protect cells from cell death associated with mitotic catastrophe (**Figure 2**).

## 4 Renal tubular injury in typical DKD and Notch

Common features of fibrosis-related renal function decline in DKD include tubular epithelial cell dedifferentiation, immune cell influx, and myofibroblast activation (57). The early manifestations of DKD are hyperfiltration and renal hypertrophy, and proximal tubule cell (PT) loss is the main cause of renal hypertrophy and hyperfiltration (58). The late stage of DKD is characterized by PT cell dedifferentiation, and the loss of renal PT cells is associated with a decrease in eGFR. Under high glucose stimulation, the expression of Polo-like kinase 2 (PLK2) increased in the kidney tissue of DKD mice. Silencing PLK2 significantly inhibited the activation of the Notch1 signaling pathway and reduced the expression of renal fibrosis-related markers, while overexpression of HES1 rescued the downregulation of markers induced by si-PLK2. PLK2 can mediate tubulointerstitial fibrosis in DKD by activating the Notch1 signaling pathway (59). Renal tubular fibrosis is considered to be a complex and irreversible metabolic change and one of the key signs of disease progression (60, 61). In the study of diabetic renal tubular fibrosis, multiple signaling pathways interact, including TGF-β, Wnt/β-catenin, MAPK, Notch, etc., forming a complex signal transduction network (61). The TGF-β pathway is considered to be the key to renal fibrosis, inducing extracellular matrix (ECM) accumulation by activating Smad proteins and non-Smad pathways. In renal fibrosis, the interaction between the TGF-β and Notch signaling pathways is essential (55). Notch signaling is activated through ADAM10-mediated proteolytic reactions, and the ADAM10-Notch signaling axis is associated with renal fibrosis (62). Ligand-receptor binding triggers Notch signaling, which results in the release of NICD. NICD translocation to the nucleus controls the expression of target genes, which includes involvement in the creation of the extracellular matrix (ECM) and EMT. In renal cells, the TGF-β pathway can upregulate Notch ligands, including Jagged1, which increases Notch activation. Notch target gene Hes-1 is also a direct downstream gene of the TGF-β pathway. The degree of tubulointerstitial fibrosis is strongly associated with NICD, which has the ability to directly affect downstream Smad3 (63). The Jumonji domain containing-3 (JMJD3) reduces the level of H3K27me3 and inhibits the activation of TGF-β and NOTCH signaling, thereby exerting an anti-renal fibrosis effect (64). Strategies that target either process (e.g., γ-secretase inhibitors or particular pathway inhibitors) have demonstrated potential for ameliorating renal fibrosis in experimental animals (65, 66). In rats with tissue fibrosis, TGF-β inhibitors decrease the expression of Notch and its target genes, Notch1, Hes1, and Hes5. By suppressing TGFβRII and Smad3, epigallocatechin gallate (EGCG) blocks the Notch pathway in renal cells (67, 68). By blocking the Notch pathway’s activation, TGF-β production, and Smad2 and Smad3 phosphorylation, Notch inhibitors can markedly lessen the severity of renal fibrosis.

**Figure 3:**
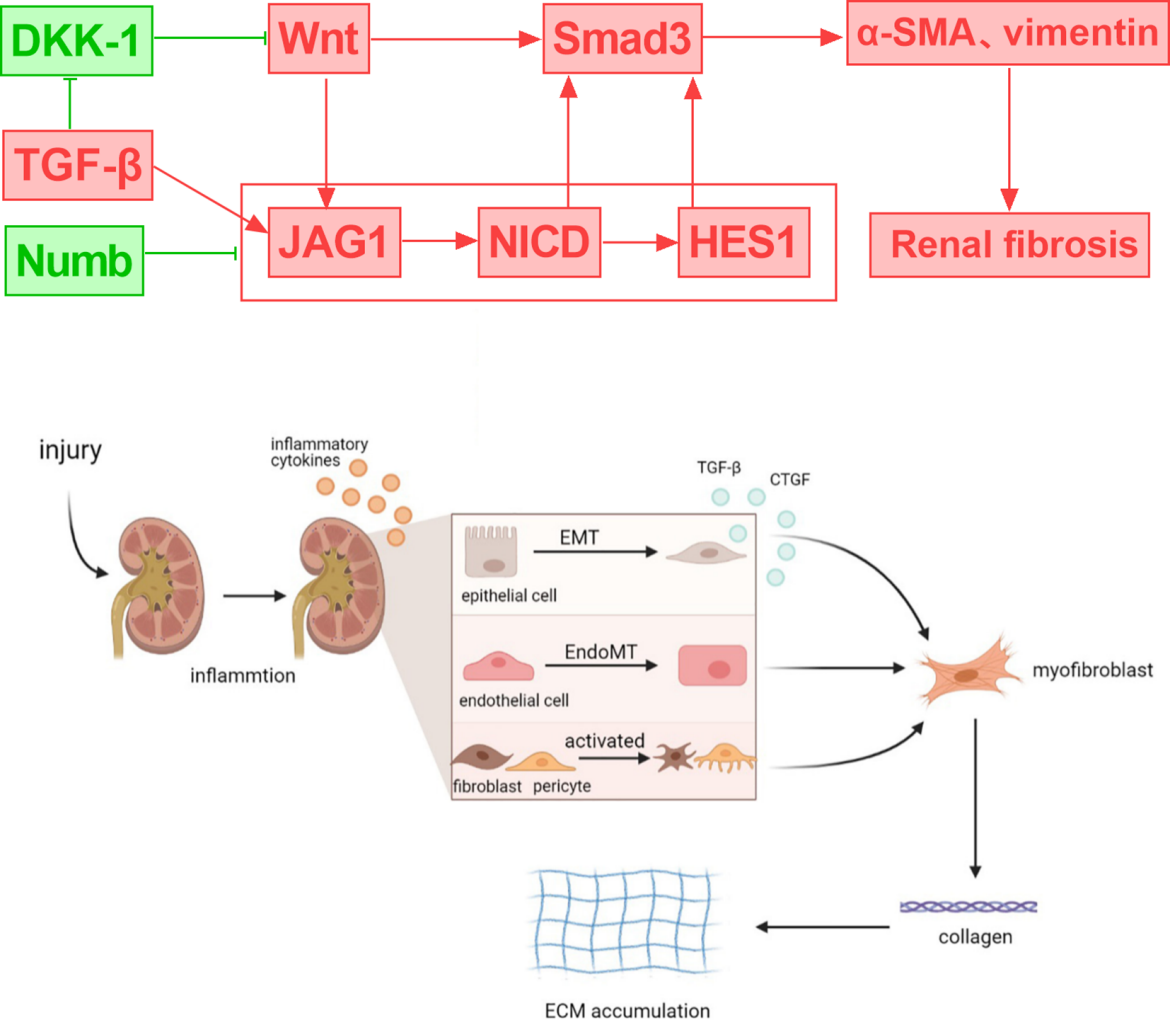
Crosstalk between notch signaling and DKD-related signals and their role in regulating renal inflammation and fibrosis.

Numb is downregulated in diabetic nephropathy tissues and high glucose-stimulated endothelial cells, while Notch1 and Hes1 are upregulated. Numb affects endothelial-mesenchymal transition (EndoMT) by negatively regulating the Notch signaling pathway, which leads to a decrease in the expression of endothelial cell markers (such as E-cadherin and CD31) and an increase in the expression of mesenchymal cell markers (such as α-SMA and vimentin), thereby participating in the pathological process of DKD and mediating renal fibrosis and disease progression (69). In addition, studies have found that the Wnt/β-catenin signaling pathway is an upstream mediator of the Notch signaling pathway. Inhibition of Wnt reduces JAG1 expression, while Wnt10b can promote the activation of Wnt and Notch signals (70–73). Downregulation of A cluster-Homeobox genes encoding a5 protein (HOXA5) by DNA methylation induces NOTCH activation and promotes renal fibrosis. HOXA5 inhibits the transcription of Jag1 by directly binding to its gene promoter, inhibiting Notch signaling and alleviating renal tubular fibrosis (74). Therefore, understanding the interplay between Notch and multiple signaling pathways could help inform potential therapeutic interventions for the management of fibrotic kidney diseases (**Figure 3**).

In the development of diabetic nephropathy, a variety of inflammatory cells participate and play an important role, especially in the process of renal fibrosis (75). Damaged intrinsic renal cells in diabetic nephropathy attract monocytes and macrophages to the site of tissue injury in order to prevent and remove cell damage. Polarization of macrophages in diabetic nephropathy was caused by interaction between the Notch system and NF-κB signaling in macrophages. Numerous inflammatory cytokines are secreted by M1 phenotype macrophages, which further worsened the intrinsic kidney cells’ fibrosis, necroptosis, extracellular matrix production, and inflammatory response (8). Inhibition of the macrophage Notch pathway can alleviate the pathological changes of renal cells. In addition to being a key regulator of kidney development and being silenced afterwards, Notch signaling is reactivated in kidney injury and is involved in acute and chronic kidney injury. *In vivo* studies have shown that Notch activation is associated with interstitial fibrosis and glomerulosclerosis (76). In the glomeruli and tubules of patients with chronic kidney disease, expression of both Notch receptors and ligands increases renal injury.

## 5 Roles of Notch-related miRNA in DKD

miR-124a can promote the differentiation of bone marrow mesenchymal stem cells into islet-like cells. Bone marrow mesenchymal stem cells combine with miR-124a to regulate specific transcription factors and genes and inhibit the activity of Notch signaling, exerting significant anti-fibrosis and repair effects, thereby effectively alleviating the progression of diabetic nephropathy (77). By blocking the Notch pathway, miR-135a inhibition can lessen renal fibrosis in DKD rats (78). Through its targeting of MAML1 and inactivation of the Notch signaling pathway, miR-133a-3p reduces the damage caused by high hyperglycemia to human renal tubular epithelial cells and prevents the progression of DKD (79). In podocytes exposed to high hyperglycemia, overexpression of miR-34c suppresses Notch signaling by specifically targeting Notch1 and Jaggged1 (80). Increased expression of miR-34a reduced podocyte damage and NICD, Hes1, Hey1, Jagged1, and Notch 1 protein expression under high glucose circumstances (81). MiR34a further enhances the effect of mesenchymal stem cell microvesicles on mouse renal fibrosis by regulating EMT and Notch pathways (82). miR-34a may be a candidate molecular therapeutic target for the treatment of renal fibrosis. Loss of miR-146a in renal tubular cells is associated with an increased risk of DKD. miR-146a normally inhibits the expression of Notch1 and ErbB4 mRNA by binding to their 3’ UTR region. When miR-146a expression is reduced or absent, this inhibitory effect is lost, resulting in increased transcription levels of Notch1 and ErbB4. Upregulation of Notch1 and ErbB4 leads to activation of the EGFR pathway, which further promotes tubular cell damage and disease progression (83).

Exosomal miR-30a-5p significantly promoted the proliferation and migration, and reduced apoptosis of glomerular endothelial cells under high glucose conditions and reduced the mRNA and protein expression levels of Notch1 and VEGF. Exosomal miR-30a-5p inhibits EMT and abnormal angiogenesis of glomerular endothelial cells by regulating the Notch1/VEGF signaling pathway. One possible therapeutic approach for DKD treatment might be miR-30a-5p (84). Targeted administration of miRNA-30a via engineered nanoplexes to save dying podocytes in DKD. miRNA-30a is primarily responsible for podocyte homeostasis. In DKD, miRNA-30a is directly and predominantly inhibited by hyperglycemic kidney-induced Notch signaling, leading to podocyte damage and apoptosis. The nanocomplex can upregulate the expression level of miRNA-30a in high glucose-exposed podocytes, significantly inhibit Notch1 signaling in diabetic C57BL/6 mice, and reduce glomerular expansion and glomerular fibrosis (85).

## 6 Clinical use of Notch signaling in DKD

In diabetic nephropathy, high concentrations of glucose promote fibrosis through the Notch1 pathway. Natural flavonoid ombuin significantly down-regulated the expression of TGF-β1, Notch 1, and Hes 1 and up-regulated the expression of peroxisome proliferator-activated receptor γ (PPAR γ), significantly improved renal function and pathological damage in DKD rats, and improved renal interstitial fibrosis. Ombuin may exert anti-inflammatory and anti-fibrotic effects by inhibiting Notch 1 activity and activating PPARγ (86). Under the Danggui-Shaoyao-San (DSS) treatment group, there were substantial decreases in the protein and mRNA levels of Jagged1, Notch1, Hes5, and NICD and significant increases in the protein and mRNA levels of E-cadherin. By blocking Notch signaling, DSS inhibits renal tubular EMT and prevents diabetic nephropathy (87). Trichostatin A (TSA) does not affect the phosphorylation levels of Smad2, Smad3, p38, and ERK but significantly reduces the phosphorylation of JNK, thereby inhibiting the activation of the Notch-2 signaling pathway. This effect leads to the downregulation of fibrosis markers such as α-SMA and fibronectin stimulated by TGF-β1, thereby alleviating the occurrence and development of renal fibrosis. By interfering with the JNK/Notch-2 signaling pathway, TSA shows potential therapeutic effect in the treatment of renal fibrosis (–).-Epigallocatechin gallate (EGCG), as a natural antioxidant, can inhibit the expression of Notch1 and reduce the activation of the TGF-β/Smad3 signaling pathway, thereby alleviating renal fibrosis in diabetic mice (88). In addition, EGCG treatment can significantly reduce the levels of Notch1 and TGF-βRII in HEK293 cells stimulated by high glucose, further supporting the potential of EGCG in the treatment of diabetic nephropathy. Therefore, the Notch signaling pathway is an important target for the treatment of fibrosis, and EGCG may alleviate related diseases by regulating the Notch pathway (67).

Baicalin inhibits the activation of the Notch1-Snail axis in podocytes, alleviates glomerular structural destruction and dysfunction, and reduces proteinuria. Baicalin is a new renal protective agent against podocyte EMT (89). Under a high glucose environment, C-peptide significantly reduces EMT and renal fibrosis by reducing the expression of Snail, Vimentin, α-SMA, and CTGF. C-peptide also inhibits the activation of Notch1 and Jagged1 in the Notch signaling pathway and TGF-β1 in the TGF-β signaling pathway, thereby alleviating the progression of diabetic nephropathy (90). Gliquidone inhibits the expression of proteins associated with the Notch signaling system, including Jagged1, Notch1, Hes1, and Snail1, hence delaying the EMT process of renal tubular epithelial cells. Gliquidone has high therapeutic promise and slows the evolution of diabetic nephropathy by inhibiting the activation of the Notch/Snail signaling pathway (91). Glucagon-like peptide-1 agonist combined with crocin treatment can inhibit Notch signaling and mesangial growth in animal models of diabetic kidney disease and significantly improve renal function (92). In the kidney tissue of model rats, the Traditional Chinese medicine (TCM) capsule for qi replenishment, yin nourishment, and blood activation can lower Hes1, CD34, and CD144, safeguard kidney function, and postpone the onset of DKD (93). By triggering autophagy and blocking the Notch pathway to reduce podocyte dedifferentiation, dasatinib and quercetin prevent DKD (94).

High glucose environments activate the Notch pathway, leading to oxidative damage of renal tubular epithelial cells and renal interstitial fibrosis. Studies have shown that the Notch pathway affects cell apoptosis by regulating mitochondrial function and related genes (such as Drp1 and PGC-1α) under high glucose conditions. Inhibitors of the Notch pathway, such as DAPT, can reduce these negative effects. GH treatment will trigger an increase in the expression of EMT markers (vimentin, SMA, etc.), while DAPT effectively prevents these changes induced by GH (95). DAPT treatment not only reduced the release of cytokines but also prevented the thickening of the renal tubular basement membrane, proteinuria, and decreased renal function caused by GH (96). Therefore, DAPT significantly improved GH-induced EMT and its related renal injury by blocking the Notch1 signaling pathway. Following therapy with DAPT, blood urea nitrogen and creatinine levels were dramatically lowered. By inhibiting the Notch signaling system, DAPT dramatically lowers the expression of Notch signaling components in renal tissue, including Jagged1, Notch1-3, and Hes1. This lessens kidney damage in diabetic rats. With its ability to suppress the Notch signaling system, DAPT offers fresh promise as a therapy for kidney damage caused by diabetes. Trichosanthes kirilowii lectin (TKL) can prevent macrophage polarization from the M2 (anti-inflammatory) to the M1 (pro-inflammatory) phenotype by inhibiting the Notch signaling pathway, reducing the expression of Notch1, NICD1, and Hes1 to inhibit Notch signaling activity, and reducing kidney damage in rats with diabetic nephropathy (97). SIRT1 promotes macrophage polarization toward the M2 phenotype by inhibiting the NOTCH signaling pathway (98). Regulating macrophage polarization through Notch signaling is an important direction for treating diabetic nephropathy.

According to research on animals, the intrauterine diabetes environment hinders the differentiation of progenitor cells into nephrons, potentially through interference with the Notch and Wnt/β-catenin signaling pathways (99). Research has shown that p66Shc activates the Notch-PTEN-PI3K/Akt/mTOR signaling pathway to cause apoptosis and block podocyte autophagy, two actions that may be useful in the management of diabetic kidney disease (DKD) (100). The human renal tubular epithelial cells (HK-2 cells) exhibit a considerable activation of their Notch1 and Hes-1 signaling pathways during high glucose stimulation, which therefore results in a reduction in autophagy levels. Autophagy activity was restored when Sirt3 overexpression was achieved using pCMV-Sirt3 transfection. This resulted in a considerable inhibition of the Notch1/Hes-1 pathway activation (101). Based on this, it appears that Sirt3 protects autophagy in HK-2 cells and might be a target for diabetic nephropathy therapy (**Table 1**).

**Table 1:** Clinical use of Notch signaling in DKD.

Involved drugs	Involved signaling pathway	Clinical effect
Flavonoid ombuin	TGF-β1↓ Hes1↓ notch1↓PPAR γ↑	anti-inflammatory and anti-fibrotic
DSS	notch1↓ Hes5↓	inhibits renal tubular EMT
TSA	JNK↓ notch2↓ TGF-β1↓	anti-fibrotic
EGCG	TGF-βRII↓ notch1↓	anti-fibrotic
Baicalin	notch1↓ Snail1↓	inhibits podocyte EMT
C-peptide	TGF-β1↓ notch1↓	reduce EMT and renal fibrosis
Gliquidone	notch1↓ Hes1↓ Snail1↓	inhibits renal tubular EMT
Glucagon-like peptide-1 agonist crocin	notch1↓	inhibit mesangial growth improve renal function
Capsule for replenishing qi, nourishing yin	notch1↓ Hes1↓	reduced 24-h urinary albumin improve renal function
Dasatinib and quercetin	notch1↓	activate autophagy and alleviate podocyte dedifferentiation
DAPT	notch1↓ Vimentin↓ SMA↓	inhibits renal tubular EMT reduce kidney damage
TKL	notch1↓ Hes1↓	prevent macrophages from M2 phenotype to M1 phenotype reduce kidney damage
p66Shc	notch1↑ mTOR↑	cause apoptosis and block podocyte autophagy
Sirt3	notch1↓ mTOR↓	protect autophagy and reduce apoptosis

“↑” mean signal upregulation.

“↓” mean signal downregulation.

## 7 Discussion

DKD is the main driver of death in diabetic patients. Controlling blood sugar alone is not enough to eliminate diabetic complications and improve survival (102). Drug treatment should focus on preventing complications rather than simply lowering blood sugar. SGLT2 is only expressed by PT cells, and SGLT2 inhibitors (SGLT2i), drugs targeting PT cells, have achieved good results. In addition to reducing heart failure mortality and mortality, they can also reduce comprehensive renal outcomes by 40% (103–105). Combination therapy such as SGLT2i and angiotensin-converting enzyme inhibitors has non-overlapping synergistic effects and can reduce PT cell damage, which suggests that combination therapy is the trend of the future (106). However, there are currently no approved drugs targeting podocytes. Current drugs primarily target renal PT cells and have been associated with GFR preservation. Future research will examine the potential therapeutic benefits of targeting different cell types, such as fibroblasts, immune cells, or podocytes.

The Notch signaling pathway regulates kidney development, angiogenesis, glomeruli, tubules, renal interstitium, macrophage function, etc. It is involved in multiple processes such as glomerular endothelial dysfunction, filtration barrier damage, podocyte EMT and dedifferentiation, tubulointerstitial fibrosis, PT cell dedifferentiation, macrophage polarization, etc. in DKD and is closely related to the onset of DKD. Notch signaling is involved in the entire process of DKD. It may become a reality to develop drugs targeting different types of kidney cells using NOTCH signaling, and combined treatment of multiple cell targets may be expected to improve the future of DKD patients. In summary, although there are still some issues to be resolved, we believe that correcting notch signaling abnormalities will become a new therapeutic strategy for DKD.
